# Otology

**Published:** 1897-12

**Authors:** W. H. Harsant


### OTOLOGY.

Among all the varieties of chronic deafness, probably the most intractable from a therapeutical point of view is **the dry form of chronic catarrh of the middle ear,** accompanied by adhesions of the ossicles. Any new method of treatment of this disease holding out a prospect of favourable results ought to be received therefore with due thankfulness, and should be afforded a thorough trial.

A remarkable suggestion in a new direction has been made by Dr. Cohen-Kysper,[1] of Hamburg, and we shall anxiously wait for further developments of his most original method of treatment. He points out that the pathological changes which, in chronic catarrh of the tympanum, may lead to permanent hardness of hearing frequently consist of a hyperplasia of the mucosa, due to epithelial deposits and proliferation of connective tissue, through which the functions of the ossicles are restricted. Analogous to catarrhal affections of other mucous membranes, this may terminate in a degenerative stage, leading to shrinking, hardening, and thickening. A condition frequently found is one marked by the formation of bands and membranes due to adhesions between portions of the hyperplastic membrane in the narrow spaces of the middle ear. This form of chronic catarrh, one of the most common forms of incurable deafness, is designated as dry catarrh, otitis media adhesiva, and frequently as sclerosis.

Dr. Cohen-Kysper attempts to affect these usually irreparable processes by introducing into the middle ear solutions of ferments having the power to dissolve albumen, for the purpose of inducing a process of digestion of the hyperplastic material, as well as of the connective tissue products; to dissolve them or lessen their size, and thereby render the conduction of sound more easy. That organised tissue *in vivo* is capable of being digested is sufficiently well known. Thus rabbits' ears and frogs' legs have been introduced into dogs' stomachs through gastric fistulæ and completely dissolved. In the middle ear we have conditions particularly favourable for the action of digestive ferments.

Various ferments have been tested. For one whole year experiments were made with the vegetable ferment papayotin. Finally, a definite and completely satisfactory solution for injection resulted from the work of Klug,[2] of Buda-Pesth. He discovered a method, as simple as it was exact, of measuring by photometry the energy of digestive ferments, and of making exact estimates of the concentration of the pepsin and of the hydrochloric acid and the differences in the pepsin from different

[1] *Arch. Otol.*, 1897, xxvi. 158.
[2] *Arch. f. d. ges. Physiol.*, 1895, lx. 43; cited by Cohen-Kysper.

animals. He found that the pepsin of the carnivorous dog far exceeded in intensity as well as rapidity of digestive power that of the herbivorous cattle and the omnivorous swine. With reference to the concentration of the solution, it was found advisable to use a concentration of .15 per cent. The percentage of hydrochloric acid should be .6 per cent.

With reference to the mode of injection into the tympanum, the always uncertain method by way of the tube was at the outset excluded, and an injection was made by a needle through the drum membrane. The injection should be made as near as possible to the stapes—that is, posterior to the manubrium mallei, in the vicinity of its upper half, or between the rim of the drumhead and the descending limb of the incus, if this can be seen through the membrane and there is sufficient space to make the puncture. The amount of injected fluid, according to experiments on the cadaver, which is sufficient to cover the stapes is from one-half to one decigramme. It is advisable to warm the solution, taking care to avoid too high a temperature for fear of destroying the pepsin. The position of the patient should be lying with the head on a low pillow and face turned to the opposite side. After the injection the patient should remain in this position for about half an hour. The instrument employed is a Koch syringe, modified to meet the requirements of ear instruments. The cannula is bent at an angle, the needle toward the point as thin as possible, the bevel short ; a piece of India-rubber tubing between the bulb and glass cylinder is indispensable to steady and safe handling. The bulb should be compressed by an assistant, who should watch the flow of the solution in order that the air following does not drive the fluid out of the tympanum.

Although simply an injection, the procedure is always delicate, often fairly difficult. It is of prime importance that a first injection be successful, as in case of failure a repetition cannot be made at once owing to the danger of excessive irritation. It should not be done again before the lapse of several months. The use of an anæsthetic is often advisable, as the pain is frequently quite severe.

It is unquestionable that an actual digestion of cicatricial tissue takes place. It is scarcely possible to explain in any other way the fact that in such cases immediately after the end of the procedure the hearing improves fivefold, and, above all, remains so. Within one-half to one hour all the result possible is usually reached. A total of 150 cases of chronic catarrh were treated, of which 40 were bilateral. Eighty cases were treated with injections of pepsin solution; in the remainder other forms of ferment were used. The number improved was more than two-thirds of the total number treated.

W. H. Harsant.

